# Plant Growth Promoting Rhizobacteria and Silicon Synergistically Enhance Salinity Tolerance of Mung Bean

**DOI:** 10.3389/fpls.2016.00876

**Published:** 2016-06-17

**Authors:** Sajid Mahmood, Ihsanullah Daur, Samir G. Al-Solaimani, Shakeel Ahmad, Mohamed H. Madkour, Muhammad Yasir, Heribert Hirt, Shawkat Ali, Zahir Ali

**Affiliations:** ^1^Department of Arid Land Agriculture, King Abdulaziz UniversityJeddah, Saudi Arabia; ^2^Department of Environmental Sciences, King Abdulaziz UniversityJeddah, Saudi Arabia; ^3^King Fahd Medical Research Center, King Abdulaziz UniversityJeddah, Saudi Arabia; ^4^Center for Desert Agriculture, King Abdullah University of Science and TechnologyThuwal, Saudi Arabia

**Keywords:** water relation, stomatal conductance, photosynthetic pigments, salt tolerance index, rhizobacteria characterization-identification

## Abstract

The present study explored the eco-friendly approach of utilizing plant-growth-promoting rhizobacteria (PGPR) inoculation and foliar application of silicon (Si) to improve the physiology, growth, and yield of mung bean under saline conditions. We isolated 18 promising PGPR from natural saline soil in Saudi Arabia, and screened them for plant-growth-promoting activities. Two effective strains were selected from the screening trial, and were identified as *Enterobacter cloacae* and *Bacillus drentensis* using matrix-assisted laser desorption ionization-time-of-flight mass spectrometry and 16S rRNA gene sequencing techniques, respectively. Subsequently, in a 2-year mung bean field trial, using a randomized complete block design with a split-split plot arrangement, we evaluated the two PGPR strains and two Si levels (1 and 2 kg ha^−1^), in comparison with control treatments, under three different saline irrigation conditions (3.12, 5.46, and 7.81 dS m^−1^). The results indicated that salt stress substantially reduced stomatal conductance, transpiration rate, relative water content (RWC), total chlorophyll content, chlorophyll a, chlorophyll b, carotenoid content, plant height, leaf area, dry biomass, seed yield, and salt tolerance index. The PGPR strains and Si levels independently improved all the aforementioned parameters. Furthermore, the combined application of the *B. drentensis* strain with 2 kg Si ha^−1^ resulted in the greatest enhancement of mung bean physiology, growth, and yield. Overall, the results of this study provide important information for the benefit of the agricultural industry.

## Introduction

Salinity in agriculture is a global problem (Al-Karaki, 2006). Owing to low rainfall and high evaporation in arid and semi-arid regions, salinity generates a low water potential in the soil, making it difficult for plants to acquire water, and resulting in water deficit conditions (Mahajan and Tuteja, 2005; Porcel et al., 2012). Salinity induces osmotic stress and ionic toxicity that leads to secondary oxidative stress in plants (Ding et al., 2010; Ahmad et al., 2016). Thus, salinity negatively affects the physiology, growth, and yield of crops (Haghighi and Pessarakli, 2013; Liu et al., 2015).

Numerous studies have reported the beneficial effect of silicon (Si) on crop growth and yield for plants under biotic and abiotic stress, but variable results have been reported for the beneficial effects of Si accumulation in plant tissues among plant species (Hodson et al., 2005; Ma and Yamaji, 2008; Abdel Latef and Tran, 2016). In various laboratory and greenhouse experiments, Si decreased the uptake of sodium (Na^+^) and chloride (Cl^−^) ions in saline conditions (Abbas et al., 2015; Garg and Bhandari, 2015). In contrast, in soil and hydroponic culture, Si improved crop growth, and yield under various stresses (Haghighi and Pessarakli, 2013; Kochanová et al., 2014; Yin et al., 2014; Wang et al., 2015). Although studies on the foliar application of Si have been conducted, no systematic research has explored its effects on physiology, growth, and yield of legume crops under saline stress in field conditions.

Bacterial inoculants have recently been used for the amelioration of salt stress in crop plants (Saravanakumar and Samiyappan, 2007; Yue et al., 2007). Plant-growth-promoting rhizobacteria (PGPR) are beneficial free-living soil-borne bacteria that colonize plant roots and improve plant growth through multiple mechanisms (Patten and Glick, 2002; Vessey, 2003). PGPR can improve plant growth via biological nitrogen fixation, biosynthesis of phytohormones, nutrient solubilization, nutrient uptake, and host plant resistance to biotic and abiotic stresses (Kang et al., 2009; Richardson et al., 2009; Kang S. et al., 2014). In addition, PGPR inoculation improves the plant growth and yield of various crops under normal and stress conditions (Arruda et al., 2013; Barnawal et al., 2014; Martínez et al., 2015). The optimal PGPR for plant protection are those isolated from the local environment (Zhang et al., 2004; Ahmad et al., 2013). Therefore, in the present study, we isolated PGPR from plants growing in saline conditions in Saudi Arabia, where natural abiotic stresses (e.g., salt, drought, and high temperatures) affect plants.

Mung bean is an important pulse crop that grows optimally in drought conditions, but is negatively affected by salinity (Ahmad et al., 2013). Thus, to effectively grow mung bean in arid conditions, where drought and salinity generally co-exist, the combined application of Si and PGPR was explored as a possibility for improving different aspects of the crop under saline conditions.

## Materials and methods

### Isolation of rhizobacteria

The PGPR were isolated from the rhizosphere of mung bean growing at the King Abdulaziz University's agricultural research station (Hada Al-Sham near Jeddah, Kingdom of Saudi Arabia), as described by Piromyou et al. (2011). A dilution plating technique with Luria Bertani (LB) agar medium was used for the isolation of rhizobacteria (Camargo et al., 2003). Soil samples (10 g) were added to 95 mL of sterile water, and the resulting suspensions were serially diluted (10^−8^). Aliquots (1 mL) were inoculated on plates containing LB agar medium. The plates were incubated for 48 h at 27°C, and bacterial colonies were differentiated by their morphology, pigmentation, and growth rate. Fast-growing colonies of bacteria were selected and purified by streaking on a fresh agar plate of LB media. Pure cultures of the strains were preserved in glycerol at -40°C for subsequent use. The rhizobacterial strains were coded (P1–P18).

### Screening of rhizobacteria

The 18 rhizobacterial isolates were screened for plant growth promoting activities in mung bean (cultivar NFM-12-9) under axenic conditions. An inoculum for each strain was prepared in sterilized conical flasks using LB broth. Each flask, containing 60 mL of sterilized LB broth, was inoculated with a selected bacterial strain and incubated for 72 h at 28 ± 1°C under shaking (100 rpm) conditions. Prior to seed inoculation, an optical density of 0.5 at 535 nm was achieved by dilution (10^8^–10^9^ colony forming units mL^−1^). Mung bean seeds were surface sterilized as described by Torres et al. (2013). Three surface-sterilized seeds were dipped into the inocula for 10 min and placed in a Jiffy-7® autoclave (Jiffy Products International AS, Norway). The experiment was a completely randomized design with replicates in triplicate. Sterilized 0.5 × Hoagland solution was used to supply water and nutrients to the plants. After 3 weeks, root and shoot growth parameters were recorded. The two most effective strains, based on their plant growth promotion of mung bean, were selected for field evaluation.

### Characterization of rhizobacteria

IAA (Indole acetic acid) production, in the presence or absence of its precursor l-tryptophan, was determined for the selected rhizobacteria, using the protocol described by Sarwar et al. (1992). Exopolysaccharide synthesis was determined following the method described by Ashraf et al. (2004), and siderophore activity was assayed as described by Schwyn and Neilands (1987). The phosphate solubilization ability of PGPR was observed in the medium, following the methods described by Mehta and Nautiyal (2001). Gram's test was performed as described by Vincent (1970), and 1-aminocyclopropane-1-carboxylate (ACC) deaminase activity was determined using the method described by Penrose and Glick (2003).

### Identification of selected rhizobacteria strains

The selected purified isolates were identified using matrix-assisted laser desorption ionization-time-of-flight mass spectrometry (MALDI-TOF MS; Bruker Daltonics, Billerica, Mass., U.S.A.), and matrix solution, in triplicate, as described by Angelakis et al. (2014). The automatic acquisition of bacterial spectra was performed using the Flex Control 3.0 software, and the analysis was done using Biotyper 2.0 software. The threshold score for identification was considered ≥1.9 (Angelakis et al., 2014). Strains that were not identified by MALDI-TOF MS were subjected to 16S rRNA gene sequencing, following the procedure described by Yasir et al. (2009). Briefly, genomic DNA was extracted from the isolate using 5% Chelex-100 solution and boiled for 20 min in 1.5 mL tubes. The supernatant was used as the template for the polymerase chain reaction (PCR). The PCR amplification was performed for the 16S rRNA gene using universal primers 27F (5′-AGAGTTTGATCMTGGCTCAG-3′) and 1492 R (5′-GGTTACCTTGTTACGACTT-3′), and using the following conditions: 94°C for 5 min (1 cycle); 94°C for 45 s, 55°C for 45 s, and 72°C for 1 min (30 cycles); and 72°C for 10 min (1 cycle). The purified PCR product was sequenced through Sanger sequencing technology, using the ABI prism sequencer 3730 (Applied Biosystems, USA), following the manufacturer's protocol. To identify the related strain, the obtained sequence was subjected to a BLAST search using the EzTaxon server (http://eztaxon-e.ezbiocloud.net/). The sequence was aligned using ClustalX, and a phylogenetic tree was constructed by a distance (neighbor-joining) method using Mega4 software (Tamura et al., 2013).

### Field trials

A 2-year (2014–15 and 2015–16) field trial was conducted at agricultural research station, Hada Al-Sham (21°48′3″ N, 39°43′25″ E), Jeddah, Saudi Arabia under natural saline conditions to evaluate the performance of the selected PGPR strains and Si applications. Prior to the start of the experiment, soil samples were taken from the experimental site and analyzed for their physical and chemical properties (Table 1), according to the methods described by Ryan et al. (2001). The experiment was a randomized complete block design with a split-split plot arrangement of four replicates, with saline irrigation as the main plot, and Si and PGPR strains as the sub-sub plots (3 × 2 m). At the start of the experiment, a basal dose of NPK fertilizer (18:18:5), at a rate of 400 kg ha^−1^, was applied to the plants.

**Table 1:** **Physical and chemical properties of soil of the experimental site used for the field trial**.

**Feature**	**Unit**	**Value**
Sand	%	75
Silt	%	14
Clay	%	11
Textural class	–	Sandy loam
Saturation percentage	%	39
pH_*s*_	–	7.73
EC_e_	dS m^−1^	2.9
Organic matter	%	0.63
Total nitrogen	%	0.037
Available phosphorus	mg kg^−1^	5.8
Extractable potassium	mg kg^−1^	95

Fresh cultures of *Enterobacter cloacae* and *Bacillus drentensis* strains were prepared in broth media, as described above. For the inoculation of mung bean seeds, a slurry was prepared consisting of sterilized peat, a broth culture of the respective strains, and sterilized sugar solution (10%) in the ratio 5:4:1 v/v (Ahmad et al., 2012). Subsequently, mung bean seeds were coated with the slurry at a rate of 50 mL kg^−1^. For the uninoculated control, seeds were coated with a similar mixture containing sterilized broth, but without bacteria. In the field, seed spacing was 30 cm row-to-row and 20 cm plant-to-plant. The crop was irrigated for 10 min each morning with saline water of EC 3.12, 5.46, and 7.81 dS m^−1^, using automatic control drip irrigation. At the research station, two sources of water were available with a salinity of EC 3.12 and 7.81 dS m^−1^, and saline water with EC 5.46 dS m^−1^ was prepared by mixing the two sources. Si (0, 1, and 2 kg ha^−1^) was applied to the foliage using split doses of potassium silicate at 10 and 30 d after germination. For the Si treatments of 1 and 2 kg ha^−1^, a 0.6 and 1.2 g Si L^−1^ solution, respectively, was applied per sub-sub plot with a hand-operated sprayer. For the control treatment, pure water was sprayed. All other agronomic practices were kept uniform across the treatments throughout the experiment.

### Stomatal conductance and transpiration rates

Stomatal conductance was measured at the abaxial surface of the fully expanded leaves using a steady state leaf porometer upgraded model SC-1 (Decagon, 2011) during the morning hours. Before the measurements were taken, the instrument was calibrated to ensure accurate readings. The transpiration rate was calculated using the following equation:

$$\text{Transpiration rate }(E)=g_{s}\times \text{VPD}$$

where, *g*_*s*_, stomatal conductance; and VPD, vapor pressure deficit calculated from air temperature and leaf temperature provided by the porometer, using an online vapor pressure calculator (http://www.srh.noaa.gov/epz/?n=wxcalc_vaporpressure).

### Relative water content and electrolyte leakage

Fresh, fully developed leaves from the top of randomly selected plants were used to measure the relative water content (RWC) and electrolyte leakage (EL). The leaves were collected, sealed in plastic bags, and transported to the laboratory. After measuring the fresh weights, leaves were immersed in distilled water for 24 h in a refrigerator. Leaves were then placed over tissue paper for blotting, and the fully turgid weight was measured. Finally, the leaf samples were oven dried at 72°C for 24 h, after which the dry weight was recorded, and RWC was determined using the equation previously described by Teulat et al. (2003).

$$\text{RWC }(\%)\;=\;\frac{\text{Fresh weight}-\text{Dry weight}}{\text{Fully turgid weight}-\text{Dry weight}}\;\times \text{ 100}$$

For the measurement of EL, the leaves were cut into uniform discs with a paper puncher and transferred into test tubes containing 25 mL deionized water. The test tubes were vortexed for 10 s, and the initial electrical conductivity (EC0) of the solution was measured. The test tubes were then refrigerated at 4°C overnight and assayed for EC1. Finally, the test tubes were autoclaved at 121°C for 20 min to determine EC2, and EL was calculated using the formula described by Yang et al. (1996).

$$\text{EL }(\%)\;=\;\frac{\text{EC1}-\text{EC0}}{\text{EC2}-\text{EC0}}\;\times \;100$$

### Photosynthetic pigments

Fresh leaves were used for chlorophyll measurements, following the protocol described by Arnon (1949). Briefly, the chlorophyll was extracted by placing fresh leaf samples (0.5 g) in a shaker with 80% acetone until the leaves were completely bleached. The extract was centrifuged at 13,000 rpm for 10 min, and the supernatant was used to measure chlorophyll a (Chl a), chlorophyll b (Chl b), and carotenoid at 663, 645, and 470 nm absorbance, respectively, using a spectrophotometer.

### Plant growth, yield, and salt tolerance index

At harvest, plant growth traits (e.g., leaf area at flowering, height, dry biomass, and seed yield) were measured from three randomly selected 1 m^2^ areas in each sub-sub plot. Leaf area was measured according to Masle and Passioura (1987). Briefly, the length and maximum width of the leaf was multiplied by the mung bean leaf factor (0.84). The salt tolerance index (STI) was calculated according to Shetty et al. (1995) as:

$$\text{Salt tolerance index }(\text{STI})\;=\;\frac{\text{DWS or DWI}}{\text{DWC}}$$

where, DWI, dry weight of stressed plant; DWI, dry weight of Si treated inoculated plant; DWC, dry weight of unstressed, non-Si treated uninoculated plants.

### Data analysis

The data collected from screening and field trials were analyzed using Statistix 8.1 software. The means were compared using the LSD test (*P* ≤ 0.05; Steel et al., 1997).

## Results

### Performance of PGPR in screening trial

For most of the isolates, shoot and root fresh weights were significantly (*P* ≤ 0.05) higher for the inoculated mung bean seedlings than for the uninoculated (control) seedlings (Table 2). However, among the tested isolates, the rhizobacterial isolates P6 (*E. cloacae*) and P16 (*B. drentensis*) showed the greatest potential for promoting plant growth of mung bean seedlings.

**Table 2:** **Screening of rhizobacteria for plant growth promotion of mung bean under gnotobiotic conditions**.

**Strain**	**Shoot fresh weight (g)**	**Root fresh weight (g)**	**Shoot length (cm)**	**Root length (cm)**
Control	0.30 ± 0.035j	0.20 ± 0.013i	11.33 ± 0.721h	10 ± 0.708e
P1	0.563 ± 0.029bc	0.543 ± 0.033b	18.67 ± 0.982bc	16.67 ± 0.982a–c
P2	0.533 ± 0.047b–e	0.480 ± 0.012bc	15 ± 0.944d–g	17.33 ± 0.721ab
P3	0.463 ± 0.038c–g	0.220 ± 0.012i	16 ± 0.817c–f	16.33 ± 0.954a–c
P4	0.406 ± 0.032f–j	0.396 ± 0.039d–f	14 ± 0.944e–h	14.67 ± 0.491cd
P5	0.540 ± 0.026b–d	0.420 ± 0.018c–e	16.33 ± 0.982c–f	18 ± 0.817a
P6	0.573 ± 0.022b	0.636 ± 0.034a	22.33 ± 0.982a	17 ± 0.472ab
P7	0.430 ± 0.049e–h	0.250 ± 0.029hi	19.67 ± 1.18ab	16 ± 0.944a–c
P8	0.470 ± 0.035b–g	0.350 ± 0.032fg	18.33 ± 0.982bc	15.33 ± 0.583b–d
P9	0.530 ± 0.035b–e	0.440 ± 0.017cd	19.67 ± 0.982ab	15.33 ± 0.545b–d
P10	0.320 ± 0.046ij	0.420 ± 0.017c–e	13.33 ± 0.721f–h	10.33 ± 0.272e
P11	0.373 ± 0.02g–j	0.440 ± 0.023cd	15.67 ± 0.721c–g	15.33 ± 0.466b–d
P12	0.413 ± 0.033f–i	0.250 ± 0.012hi	16 ± 0.472c–f	15.67 ± 0.537bc
P13	0.533 ± 0.023b–e	0.250 ± 0.017hi	17.67 ± 0.272b–d	13.33 ± 0.272d
P14	0.333 ± 0.02h–j	0.300 ± 0.023gh	13.33 ± 0.721f–h	10.67 ± 0.691e
P15	0.316 ± 0.05ij	0.360 ± 0.035e–g	12.67 ± 0.272gh	15.33 ± 0.721b–d
P16	0.736 ± 0.052a	0.656 ± 0.018a	22 ± 0.817a	18 ± 0.472a
P17	0.503 ± 0.049b–f	0.320 ± 0.017g	18.67 ± 0.272bc	11 ± 0.33e
P18	0.443 ± 0.052d–g	0.420 ± 0.023c–e	17 ± 1.70b–e	13.33 ± 0.759d
LSD	0.109	0.0683	3.088	2.288

*Data represent the means ± SE of three repeats. Means having different letters are significantly different at P ≤ 0.05 according to LSD*.

Inoculation of rhizobacterial strains showed significant results for shoot fresh weight. All the tested strains increased the shoot fresh weight of mung bean over that of the control, with the highest shoot fresh weight noted for the P16 and P6 strains (Table 2). Rhizobacterial inoculation significantly increased the root fresh weight of mung bean seedlings over that of the control, except for the P3, P7, P12, and P13 strains. A marked increase in root fresh weight was noted for the P16 and P6 strains (Table 2).

Shoot length also increased significantly in mung bean plants when inoculated with rhizobacterial strains, except for the P4, P10, P14, and P15 inoculated seedlings (Table 2). The highest values for shoot length were noted for P6 and P16. Significant increases in root length were also recorded for the P2, P5, P6, and P16 strains (Table 2). However, strains P10, P14, and P17 had no significant effect on root length in mung bean seedlings.

### Characterization and identification of rhizobacteria

In the presence and absence of L-tryptophan, higher IAA production was noted for P16 than for P6 (Table 3). Exopolysaccharides production and phosphate solubilization activity were observed in both strains. In addition, P16 was able to synthesize siderophores, whereas P6 was not. Likewise, ACC-deaminase activity was noted only for P16.

**Table 3:** **Plant growth promoting characteristics of selected rhizobacterial isolates**.

**Isolates**	**IAA production (mg L**^**−1**^**)**		**ACC-deaminase (μmol mg^−1^h^−1^)**	**EPS**	**PS**	**Sid**	**Gram test**
	**Without L-TRP**	**With L-TRP**					
P16	3.86	27.53	0.17	+	+	+	+ve
P6	3.59	24.86	0.0	+	+	−	−ve

*IAA, Indole acetic acid; L-TRP, L-Tryptophan; ACC, 1-aminocyclopropane-1-carboxylate; EPS, Exopolysaccharide activity; PS, Phosphate solublization; Sid, Siderophore activity*.

**Figure 1:**
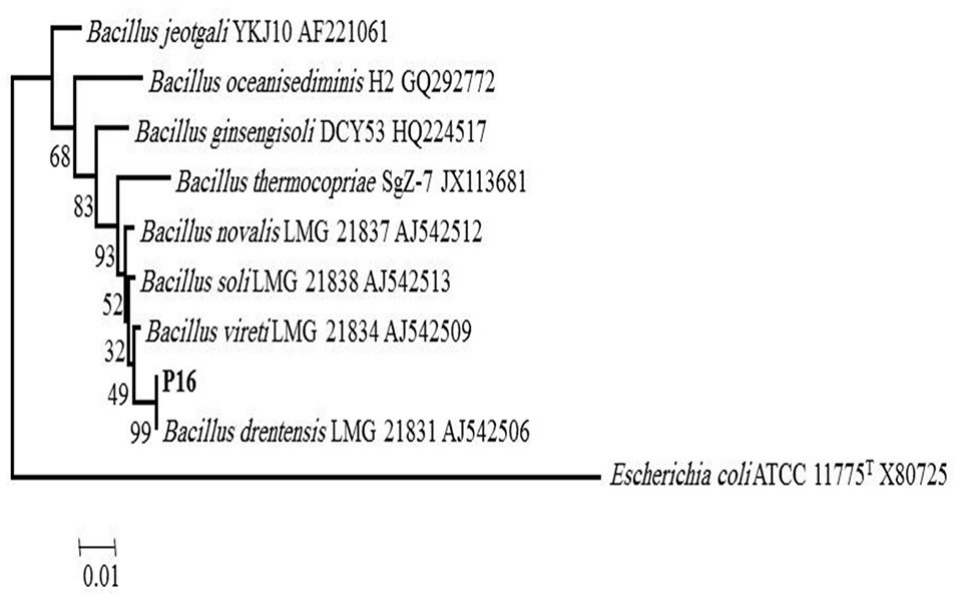
**Phylogenetic tree of P16 strain (***Bacillus drentensis***)**.

Strain P6 was identified as *Enterobacter cloacae* based on a MALDI-TOF spectral score of >1.9. Strain P16 was not identified by the MALDI-TOF technique, but the 16S rRNA gene sequence of the strain showed 100% sequence similarity with that of *Bacillus drentensis* strain LMG 21831 (AJ542506). The other closely related strains were *Bacillus vireti* and *Bacillus soli*. In the phylogenetic tree, P16 joined the cluster comprising the *Bacillus drentensis* species with 99% bootstrap support (Figure 1).

### Field trial

#### Stomatal conductance and transpiration rates

Stomatal conductance and transpiration rates decreased with increasing salinity (Table 4). On the other hand, Si and PGPR (P16 > P6) increased stomatal conductance and transpiration rates under salt stress conditions (Table 4). The 2 kg Si ha^−1^ foliar spray enhanced the stomatal conductance and transpiration rate by 42.15 and 94.67%, respectively, over that of the control (no Si spray). A higher stomatal conductance (0.394 mmol m^−2^ S^−1^) and transpiration rate (3.644 mmol m^−2^ S^−1^) were noted for the P16 treatment than for the P6 and control treatments. For these parameters, there were significant (*P* ≤ 0.01) interactions between salinity × silicon (S × Si) and salinity × PGPR strain (S × P), whereas the interaction of silicon × PGPR strain (Si × P) was not significant (*P* ≤ 0.05) for stomatal conductance, but significant (*P* ≤ 0.01) for the transpiration rate. The mean values of the S × Si interaction (**Table 7**) indicated that the highest values for stomatal conductance (0.561 mmol m^−2^ S^−1^) and transpiration rate (6.808 mmol m^−2^ S^−1^) for the 2 kg Si ha^−1^ treatment was for the 3.12 dS m^−1^ saline irrigation, followed by the 5.46 and 7.81 dS m^−1^ treatments. Similarly, for the S × P interaction (**Table 7**), the highest stomatal conductance (12.72%), and transpiration rate (29.23%) was noted for the P16 strain in the 3.12 dS m^−1^ treatment. The lowest values for stomatal conductance and transpiration rate were noted for the control treatment with the 7.81 dS m^−1^ saline irrigation. Under the same salinity level, the stomatal conductance and transpiration rate were 70.42 and 124.73% higher, respectively, in the P16 treatment than in the control treatment. The Si × P interaction (**Table 8**) indicated that, compared to the uninoculated, non-Si control treatment, the highest improvement in transpiration rate was for the P16 strain with 2 kg Si ha^−1^, followed by the P6 strain.

**Table 4:** **Effect of foliar application of silicon and bacterial inoculation on stomatal conductance (***g***_***s***_), transpiration rate (***E***), relative water content (RWC), and electrolyte leakage (EL) of mung bean under different levels of saline irrigation**.

		***g_*s*_* (mmol m^−2^ S^−1^)**	***E* (mmol m^−2^ S^−1^)**	**RWC (%)**	**EL (%)**
**SALINITY (dS m**^−1^**)**					
	3.12	0.469 ± 0.022a	4.423 ± 0.328a	83.77 ± 1.50a	12.65 ± 0.242c
	5.46	0.275 ± 0.023 b	3.271 ± 0.287b	78.97 ± 2.01b	18.19 ± 0.993b
	7.81	0.197 ± 0.009b	1.337 ± 0.084c	70.83 ± 2.39c	23.99 ± 1.30a
	LSD	0.081	0.398	3.80	1.016
**SILICON (kg ha**^−1^**)**					
	0	0.257 ± 0.020c	2.253 ± 0.181b	69.16 ± 2.32b	19.82 ± 1.37a
	1	0.319 ± 0.025b	2.391 ± 0.186b	80.42 ± 1.61a	18.53 ± 1.19b
	2	0.365 ± 0.032a	4.386 ± 0.433a	84.00 ± 1.75a	16.49 ± 1.05c
	LSD	0.025	0.212	4.77	0.609
**PGPR STRAIN**					
	Uninoculated	0.243 ± 0.027c	2.024 ± 0.269c	69.94 ± 2.24b	24.58 ± 1.47a
	P6	0.304 ± 0.025b	3.367 ± 0.320b	80.21 ± 1.60a	15.75 ± 0.580b
	P16	0.394 ± 0.021a	3.644 ± 0.343a	83.42 ± 2.01a	14.51 ± 0.598c
	LSD	0.027	0.2404	3.24	0.544
**SIGNIFICANCE**					
Salinity (S)		^**^	^**^	^**^	^**^
Silicon (Si)		^**^	^**^	^**^	^**^
PGPR Strain (P)		^**^	^**^	^**^	^**^
S × Si		^**^	^**^	^**^	^**^
S × P		^**^	^**^	ns	^**^
Si × P		ns	^**^	ns	ns
S × Si × P		ns	ns	ns	ns

Data represent the means ± SE of four replicates. Means having different letters are significantly different at P ≤ 0.05 according to LSD. ns not significant at P ≤ 0.05;

** *significant at P ≤ 0.01*.

#### Relative water content and electrolyte leakage

Significant differences (*P* ≤ 0.01) in the RWC of mung bean were observed for saline irrigation, Si, and bacterial inoculation (Table 4). Salt stress induced a marked decline in RWC, with the lowest RWC noted for the 7.81 dS m^−1^ saline treatment. Conversely, 1 and 2 kg Si ha^−1^ significantly increased the RWC by 16.28 and 21.45%, respectively, over that of the non-Si control. Similarly, bacterial inoculation had a significant effect on RWC, with the highest value noted for the P16 strain. Si and bacterial inoculation clearly increased the RWC, whereas saline irrigation decreased this parameter. In addition, there was a significant (*P* ≤ 0.01) interaction of S × Si for RWC. The highest RWC (86.23%) was noted for the 2 kg Si ha^−1^ treatment with the 3.12 dS m^−1^ saline irrigation; the lowest RWC (55.52%) was noted for the non-Si control treatment with the 7.81 dS m^−1^ saline irrigation (**Table 7**).

For EL, the individual effect of all treatments was significant (*P* ≤ 0.01; Table 4). EL increased with increasing irrigation salinity, whereas it decreased for Si (2 kg Si ha^−1^ < 1 kg Si ha^−1^) and for the strains (P16 < P6). Table 4 shows the significant (*P* ≤ 0.01) interactions of S × Si and S × P for EL. The S × Si interaction means (**Table 7**) indicated a significant reduction (24%) in EL for the 2 kg Si ha^−1^ treatment under severe salinity stress (7.81 dS m^−1^), from that of the non-Si treatment. The S × P interaction revealed the highest EL for the uninoculated control with a 7.81 dS m^−1^ salinity level, and the lowest EL for the P16 inoculation with a 3.12 dS m^−1^ salinity level. For all levels of saline irrigation, EL was significantly lower for the P16 and P6 treatments than for the uninoculated control treatment. In particular, the P16 strain repressed the negative effects of the salt stress by decreasing EL by 46.77 and 46.25% in the 5.46 and 7.81 dS m^−1^ saline treatments, respectively, from that of the control.

#### Photosynthetic pigments

Photosynthetic pigments (total chlorophyll content, Chl a, Chl b, and carotenoids) were significantly (*P* ≤ 0.01) affected by all the treatments (Table 5). An increase in salinity decreased all the photosynthetic pigments. Si and rhizobacterial inoculation increased the photosynthetic pigments, with 2 kg Si ha^−1^ and the P16 strain being the most effective (Table 5). The mean values for the S × Si and S × P interactions (**Table 7**) revealed that, for the 3.12 dS m^−1^ salinity level, the 2 kg Si ha^−1^ treatment and the P16 strain had the highest values for Chl a (0.787 g kg^−1^) and total chlorophyll (1.123 g kg^−1^), respectively. The highest reduction in Chl a and total chlorophyll content occurred under severe saline stress (7.81 dS m^−1^). However, at this salinity level, 2 kg Si ha^−1^ and P16 inoculation mitigated the adverse salt stress effects by increasing the Chl a and total chlorophyll content by 37.26 and 25.15%, respectively, over their respective control treatments. Moreover, there were significant (*P* ≤ 0.05) Si × P interactions for Chl a and Chl b, with plants treated with P16 and 2 kg Si ha^−1^ maintaining an increase of 12.67 and 51.45%, respectively, compared to the non-Si uninoculated control (**Table 8**).

**Table 5:** **Effect of foliar application of silicon and bacterial inoculation on total chlorophyll content, chlorophyll a (Chl a), chlorophyll b (Chl b), and carotenoids of mung bean under different levels of saline irrigation**.

		**Total Chlorophyll (g kg^−1^)**	**Chl a (g kg^−1^)**	**Chl b (g kg^−1^)**	**Carotenoids (mg kg^−1^)**
**SALINITY (dS m**^−1^**)**					
	3.12	1.059 ± 0.031a	0.616 ± 0.031a	0.530 ± 0.024a	3.383 ± 0.149a
	5.46	0.865 ± 0.034b	0.419 ± 0.015b	0.298 ± 0.021b	1.897 ± 0.147b
	7.81	0.756 ± 0.026c	0.312 ± 0.009c	0.224 ± 0.020b	1.226 ± 0.102b
	LSD	0.057	0.091	0.103	0.722
**SILICON (kg ha**^−1^**)**					
	0	0.765 ± 0.031c	0.358 ± 0.024c	0.250 ± 0.023c	1.851 ± 0.191c
	1	0.893 ± 0.038b	0.450 ± 0.020b	0.375 ± 0.031b	2.136 ± 0.193b
	2	1.022 ± 0.029a	0.539 ± 0.034a	0.426 ± 0.031a	2.519 ± 0.209a
	LSD	0.078	0.034	0.047	0.257
**PGPR STRAIN**					
	Uninoculated	0.786 ± 0.040c	0.398 ± 0.031c	0.266 ± 0.027c	1.893 ± 0.193c
	P6	0.910 ± 0.032b	0.454 ± 0.026b	0.357 ± 0.029b	2.142 ± 0.190b
	P16	0.984 ± 0.031a	0.495 ± 0.028a	0.428 ± 0.030a	2.471 ± 0.214a
	LSD	0.054	0.029	0.027	0.181
**SIGNIFICANCE**					
Salinity (S)		^**^	^**^	^**^	^**^
Silicon (Si)		^**^	^**^	^**^	^**^
PGPR Strain (P)		^**^	^**^	^**^	^**^
S × Si		ns	^**^	ns	ns
S × P		^**^	ns	ns	ns
Si × P		ns	^*^	^*^	ns
S × Si × P		ns	ns	ns	ns

Data represent the means ± SE of four replicates. Means having different letters are significantly different at P ≤ 0.05 according to LSD. ns, not significant at P ≤ 0.05;

* significant at P < 0.05;

** *significant at P ≤ 0.01*.

#### Plant height

Salinity considerably reduced the plant height of mung bean (Table 6). A significant (*P* ≤ 0.01) reduction in plant height was observed for increasing levels of salinity (3.12 to 7.81 dS m^−1^). In contrast, plant height significantly (*P* ≤ 0.01) increased with increasing doses of Si. Likewise, P6 and P16 inoculations significantly (*P* ≤ 0.01) increased plant height by 14.48 and 27.17%, respectively, over that of the uninoculated control. Significant (*P* ≤ 0.01) interactions were observed for S × P and Si × P, whereas the S × Si and S × Si × P interactions were not significant (*P* ≤ 0.05). The interaction of S × P revealed that, for all salinity treatments, plant height was significantly higher for P16 than for P6 and the uninoculated control (Table 7). The highest plant height (53.44 cm) was noted for plants inoculated with the P16 strain and subjected to weak salinity stress (3.12 dS m^−1^). A marked decline in plant height, 35.21 and 28.59 cm, was noted for uninoculated plants at the 5.46 and 7.81 dS m^−1^ salinity levels, respectively. Furthermore, the Si × P interaction revealed that the P16 strain with the 2 kg Si ha^−1^ resulted in the highest plant height, whereas the lowest plant height was noted for the uninoculated, non-Si plants (Table 8).

**Table 6:** **Effect of foliar application of silicon and bacterial inoculation on plant height, leaf area, dry biomass, seed yield, and salt tolerance index of mung bean under different levels of saline irrigation**.

		**Plant height (cm)**	**Leaf area (cm^2^/plant)**	**Dry biomass (t ha^−1^)**	**Seed yield (t ha ^−1^)**	**Salt tolerance index**
**SALINITY (dS m**^**−1**^**)**						
	**3.12**	**47.82 ± 0.913a**	**355.11 ± 18.96a**	**10.72 ± 0.321a**	**2.574 ± 0.121a**	–
	**5.46**	**39.01 ± 0.769b**	**286.96 ± 13.12b**	**5.16 ± 0.207b**	**1.456 ± 0.094b**	**0.601 ± 0.024a**
	**7.81**	**35.62 ± 0.981c**	**210.86 ± 12.13c**	**2.26 ± 0.098c**	**0.646 ± 0.048c**	**0.264 ± 0.011b**
	**LSD**	**0.961**	**28.06**	**0.277**	**0.224**	**0.032**
**SILICON (kg ha**^**−1**^**)**						
	**0**	**38.95 ± 1.093c**	**240.04 ± 15.00c**	**5.39 ± 0.572c**	**1.230 ± 0.128c**	**0.375 ± 0.037c**
	**1**	**40.70 ± 1.236b**	**286.34 ± 16.90 b**	**6.10 ± 0.628b**	**1.339 ± 0.131b**	**0.442 ± 0.043b**
	**2**	**42.80 ± 1.310a**	**326.57 ± 18.93a**	**6.65 ± 0.684a**	**2.107 ± 0.181a**	**0.480 ± 0.044a**
	**LSD**	**1.06**	**15.79**	**0.308**	**0.099**	**0.025**
**PGPR STRAIN**						
	**Uninoculated**	**35.84 ± 1.131c**	**202.1 ± 12.44 c**	**5.07 ± 0.542c**	**1.320 ± 0.153c**	**0.349 ± 0.035c**
	**P6**	**41.03 ± 0.783b**	**281.65 ± 11.07 b**	**5.78 ± 0.607b**	**1.530 ± 0.165b**	**0.394 ± 0.030b**
	**P16**	**45.58 ± 1.184a**	**369.18 ± 17.32 a**	**7.28 ± 0.691a**	**1.826 ± 0.157a**	**0.553 ± 0.047a**
	**LSD**	**0.948**	**14.98**	**0.351**	**0.129**	**0.0182**
**SIGNIFICANCE**						
**Salinity (S)**		^**^	^**^	^**^	^**^	^**^
**Silicon (Si)**		^**^	^**^	^**^	^**^	^**^
**PGPR Strain (P)**		^**^	^**^	^**^	^**^	^**^
**S × Si**		**ns**	^**^	^**^	^**^	^**^
**S × P**		^**^	^**^	^**^	^**^	^**^
**Si × P**		^**^	**ns**	**ns**	**ns**	^*^
**S × Si × P**		**ns**	**ns**	**ns**	**ns**	**ns**

Data represent the means ± SE of four replicates. Means having different letters are significantly different at P ≤ 0.05 according to LSD. ns, not significant at P ≤ 0.05;

* significant at P ≤ 0.05;

** *significant at P ≤ 0.01*.

**Table 7:** **Interaction between salinity and silicon; salinity and PGPR strain on growth, yield, and physiology of mung bean**.

**Salinity (dS m^−1^)**	**Silicon (kg ha^−1^)**	**Chl a (g kg^−1^)**	**RWC (%)**	**EL (%)**	***E* (mmol) *E* (m^−2^ S^−1^)**	***g_*s*_* (mmol) *m^−2^ S^−1^)***	**Leaf area (cm^2^ plant^−1^)**	**Dry biomass (t ha^−1^)**	**Seed yield (t ha^−1^)**	**Salt tolerance index**
3.12	0	0.494 ± 0.043	82.51 ± 1.83	13.11 ± 0.49	3.067 ± 0.166	0.390 ± 0.023	298.28 ± 31.11	9.746 ± 0.347	2.067 ± 0.121	–
	1	0.568 ± 0.031	82.58 ± 2.76	12.74 ± 0.35	3.396 ± 0.173	0.457 ± 0.032	356.40 ± 29.21	10.708 ± 0.549	2.208 ± 0.098	–
	2	0.787 ± 0.047	86.23 ± 3.13	12.12 ± 0.38	6.808 ± 0.435	0.561 ± 0.042	410.66 ± 32.19	11.730 ± 0.618	3.448 ± 0.111	–
5.46	0	0.323 ± 0.029	69.47 ± 2.97	19.09 ± 1.78	2.504 ± 0.310	0.217 ± 0.025	229.67 ± 15.40	4.410 ± 0.373	1.168 ± 0.149	0.512 ± 0.043
	1	0.459 ± 0.006	82.15 ± 3.02	18.85 ± 1.74	2.467 ± 0.278	0.294 ± 0.043	291.67 ± 19.29	5.406 ± 0.289	1.324 ± 0.139	0.628 ± 0.033
	2	0.475 ± 0.011	85.31 ± 2.85	16.65 ± 1.69	4.842 ± 0.527	0.316 ± 0.045	339.54 ± 21.97	5.677 ± 0.329	1.878 ± 0.134	0.662 ± 0.041
7.81	0	0.259 ± 0.013	55.52 ± 2.47	27.28 ± 2.33	1.191 ± 0.143	0.165 ± 0.009	192.16 ± 20.44	2.038 ± 0.178	0.455 ± 0.031	0.237 ± 0.021
	1	0.325 ± 0.014	76.53 ± 2.44	24.02 ± 2.16	1.311 ± 0.156	0.206 ± 0.015	210.94 ± 23.17	2.188 ± 0.152	0.487 ± 0.033	0.255 ± 0.018
	2	0.355 ± 0.005	80.46 ± 3.13	20.71 ± 2.03	1.509 ± 0.136	0.221 ± 0.016	229.50 ± 19.68	2.560 ± 0.158	0.997 ± 0.058	0.298 ± 0.019
	LSD	0.059	8.27	1.052	0.365	0.044	27.34	0.534	0.173	0.035
**Salinity (dS m**^−1^**)**	**PGPR Strain**	**Total Chlorophyll (g kg**^−1^**)**	**Plant height (cm)**	**EL (%)**	***E*** **(mmol) (m**^−2^ **S**^−1^**)**	***g***_*s*_ (mmol) (*m*^−2^ ***S*^−1^)**	**Leaf area (cm**^2^ **plant**^−1^**)**	**Dry biomass (t ha**^−1^**)**	**Seed yield (t ha**^−1^**)**	**Salt tolerance index**
3.12	Control	1.025 ± 0.053	43.73 ± 0.78	13.67 ± 0.24	3.669 ± 0.477	0.440 ± 0.045	258.66 ± 14.78	9.238 ± 0.279	2.339 ± 0.228	–
	P16	1.123 ± 0.043	53.44 ± 1.46	11.38 ± 0.35	4.868 ± 0.620	0.496 ± 0.018	479.39 ± 20.27	12.363 ± 0.598	2.728 ± 0.188	–
	P6	1.032 ± 0.064	46.29 ± 0.77	12.93 ± 0.36	4.734 ± 0.580	0.472 ± 0.047	327.28 ± 21.96	10.582 ± 0.318	2.655 ± 0.215	–
5.46	Control	0.675 ± 0.056	35.21 ± 0.81	26.19 ± 0.58	1.642 ± 0.235	0.149 ± 0.004	226.69 ± 16.33	4.340 ± 0.202	1.070 ± 0.177	0.505 ± 0.025
	P16	1.004 ± 0.037	42.24 ± 1.35	13.94 ± 0.41	4.379 ± 0.450	0.446 ± 0.025	353.88 ± 19.14	6.654 ± 0.150	1.991 ± 0.116	0.774 ± 0.021
	P6	0.917 ± 0.037	39.60 ± 0.91	14.96 ± 0.42	3.793 ± 0.391	0.232 ± 0.011	280.31 ± 16.41	4.498 ± 0.212	1.310 ± 0.128	0.523 ± 0.025
7.81	Control	0.660 ± 0.039	28.59 ± 0.66	33.90 ± 1.16	0.750 ± 0.071	0.142 ± 0.003	120.96 ± 6.89	1.658 ± 0.073	0.552 ± 0.070	0.193 ± 0.010
	P16	0.826 ± 0.047	41.08 ± 0.96	18.22 ± 0.97	1.688 ± 0.078	0.242 ± 0.009	274.27 ± 11.95	2.842 ± 0.061	0.762 ± 0.096	0.331 ± 0.009
	P6	0.782 ± 0.039	37.21 ± 0.62	19.89 ± 0.68	1.574 ± 0.096	0.208 ± 0.012	237.36 ± 7.15	2.287 ± 0.142	0.625 ± 0.076	0.266 ± 0.016
	LSD	0.088	1.642	0.943	0.415	0.047	25.95	0.608	0.223	0.025

*Means having difference greater than LSD values are significant at P ≤ 0.05*.

**Table 8:** **Interaction between silicon and PGPR strain on growth and physiology of mung bean**.

**Silicon (kg ha^−1^)**	**PGPR strain**	**Chl a (g kg^−1^)**	**Chl b (g kg^−1^)**	***E* (mmol m^−2^ S^−1^)**	**Plant height (cm)**	**Salt tolerance index**
0	Un-inoculated	0.264 ± 0.031	0.197 ± 0.037	1.489 ± 0.300	34.83 ± 2.16	0.289 ± 0.048
	P16	0.432 ± 0.041	0.293 ± 0.039	2.704 ± 0.284	41.48 ± 1.55	0.516 ± 0.073
	P6	0.380 ± 0.040	0.260 ± 0.042	2.569 ± 0.246	40.54 ± 1.39	0.319 ± 0.038
1	Un-inoculated	0.419 ± 0.044	0.268 ± 0.051	1.656 ± 0.334	36.02 ± 1.99	0.367 ± 0.064
	P16	0.477 ± 0.032	0.482 ± 0.042	2.918 ± 0.257	45.48 ± 2.09	0.553 ± 0.086
	P6	0.456 ± 0.025	0.377 ± 0.050	2.600 ± 0.272	40.61 ± 1.45	0.405 ± 0.063
2	Un-inoculated	0.513 ± 0.059	0.334 ± 0.045	2.916 ± 0.618	36.68 ± 1.83	0.392 ± 0.069
	P16	0.578 ± 0.065	0.511 ± 0.053	5.312 ± 0.773	49.81 ± 1.84	0.588 ± 0.095
	P6	0.527 ± 0.059	0.435 ± 0.052	4.932 ± 0.713	41.94 ± 1.29	0.460 ± 0.048
	LSD	0.051	0.047	0.415	1.64	0.031

*Means having difference greater than LSD values are significant at P ≤ 0.05*.

#### Leaf area

Salinity stress significantly (*P* ≤ 0.01) reduced the leaf area of mung bean (Table 6), whereas increased application of Si significantly increased leaf area. Rhizobacterial inoculation significantly increased leaf area, with a higher leaf area noted for P16 than for P6 and the uninoculated control. In addition, there were significant (*P* ≤ 0.01) S × Si and S × P interactions for the leaf area of mung bean (Table 6).

Mean values for the S × Si interaction (Table 7) suggested that the highest leaf area (410.66 cm^2^ plant^−1^) was for the 2 kg Si ha^−1^ treatment with 3.12 dS m^−1^ of saline irrigation. The lowest leaf area (192.16 cm^2^ plant^−1^) was observed for the 7.81 dS m^−1^ saline irrigation in plots where no Si was applied. Similarly, the S × P interaction (Table 7) indicated a higher leaf area for P16 inoculated plants under low saline stress (3.12 dS m^−1^), and a lower leaf area for uninoculated plants under high salinity conditions (7.81 dS m^−1^). For the different salinity treatments, 3.12, 5.46, and 7.81 dS m^−1^, leaf area increased by 85, 56, and 126% for the P16 strain and by 26, 23, and 96% for the P6, respectively, over that of the uninoculated control.

#### Dry biomass

Dry biomass production for mung bean was severely decreased by saline irrigation, whereas it increased with the application of Si and rhizobacterial inoculation (Table 6). Furthermore, significant (*P* ≤ 0.01) interactions were observed for S × Si and S × P for dry biomass production. The S × Si interaction revealed that the highest dry biomass was for the 2 kg Si ha^−1^ treatment under the low saline irrigation conditions, whereas the lowest dry biomass was observed for the 7.81 dS m^−1^ plots without Si (Table 7). Likewise, the S × P interaction showed that P16 enhanced dry biomass under all salinity stress levels, with the highest biomass (12.36 t ha^−1^) noted for P16 plants in the 3.12 dS m^−1^ treatment, and the lowest biomass (1.658 t ha^−1^) noted for the uninoculated plants in the 7.81 dS m^−1^ treatment (Table 7). In the 7.81 dS m^−1^ treatment, inoculation with the P6 and P16 strains significantly increased the dry biomass of mung bean by 38 and 71%, respectively, over that of the uninoculated plants.

#### Seed yield

Salinity significantly (*P* ≤ 0.01) decreased the seed yield of mung bean, whereas increasing rates of Si and rhizobacterial inoculation (P16 > P6) increased seed yield (Table 6). Moreover, there were significant (*P* ≤ 0.01) S × Si and S × P interactions for the seed yields of mung bean.

The S × Si interaction (Table 7) revealed that the highest seed yield (3.45 t ha^−1^) was recorded for 2 kg Si ha^−1^ under 3.12 dS m^−1^ salinity, and the lowest yield (0.46 t ha^−1^) was recorded for the non-Si control under 7.81 dS m^−1^ salinity. The 2 kg Si ha^−1^ treatment significantly improved the seed yield by 119% over that of the non-Si control, under 7.81 dS m^−1^ salinity. For all levels of salinity stress, 1 kg Si ha^−1^ had no significant effect on seed yield. The S × P interaction (Table 7) showed that P16 was a potential strain for enhancing seed yield under all levels of salinity, with maximum yield (2.72 t ha^−1^) noted for the 3.12 dS m^−1^ treatment. Saline stress at 7.81 dS m^−1^ significantly reduced seed yields, and the lowest value (0.552 t ha^−1^) was recorded for the uninoculated control.

#### Salt tolerance index

The STI was significantly (*P* ≤ 0.01) affected by salinity, Si, and rhizobacterial strains (Table 6). Moreover, S × Si, S × P (*P* ≤ 0.01), and Si × P (*P* ≤ 0.05) interactions were significant, whereas the S × Si × P interaction was not significant (*P* ≤ 0.05). The S × Si interaction (Table 7) showed that the highest STI (0.662) noted for the 2 kg Si ha^−1^ treatment with 5.46 dS m^−1^ saline stress, and the lowest STI (0.237) noted for the non-Si plots with 7.81 dS m^−1^ saline irrigation. Table 7 also shows the S × P interaction, with the P16 strain increasing STI by 35 and 71% under 5.46 and 7.81 dS m^−1^ of saline irrigation, respectively, over that of the uninoculated controls. The Si × P interaction (Table 8) revealed that the highest increase in STI (0.588) was recorded for the P16 inoculated plants with 2 kg Si ha^−1^, whereas the lowest STI (0.289) was observed for the uninoculated, non-Si plants.

## Discussion

In this 2-year field study, foliar spray of Si (1 and 2 kg ha^−1^) and two PGPR strains (*Enterobacter cloacae*, P6 and *Bacillus drentensis*, P16) were evaluated for their effect on the growth and physiology of mung bean under saline irrigation conditions. Our data suggest that the combined use of Si and PGPR is an effective approach for maximizing mung bean productivity on marginal land with saline water. The combined effects of Si and PGPR help the mung bean plants acclimatize to saline conditions by modifying a variety of plant physiological processes, including stomatal conductance, transpiration rate, water relations, and synthesis of photosynthetic pigments, thereby improving growth (plant height, leaf area, and dry biomass) and seed yield.

Currently, global efforts are underway to isolate rhizobacterial strains and to screen and characterize the strains for maximizing crop production in stress and non-stress conditions. Many bacterial genera, including *Arthrobacter, Azospirillum, Bacillus, Enterobacter, Pseudomonas, Rhizobium, Acinetobacter, Burkholderia*, etc., perform plant growth promoting (PGP) activities in different crop plants (Won-I et al., 2011; Arruda et al., 2013; Hussain et al., 2014). In the first part of our study, 18 PGPR strains were tested for PGP activities in mung bean. Among the tested strains, P16 (*Bacillus drentensis*) and P6 (*Enterobacter cloacae*) were the most promising for growth promotion (i.e., leading to the greatest improvements in root or shoot length, and root or shoot fresh weight) of mung bean. Both of these strains have IAA production, phosphate solubilization, and exopolysaccharide activity, and these traits might be the cause of the noted plant growth promotion (Zahir et al., 2004; Nadeem et al., 2010; Arruda et al., 2013; Günes et al., 2014). This potential causal relationship is supported by Arruda et al. (2013) who noted that inoculation of maize seedlings with *Achromobacter* sp. and *Burkholderia* sp., possessing IAA and phosphate solubilization activity, significantly enhanced the dry weight of root and shoot. Similar effects of PGPR inoculation on plant growth promotion have been observed by various researchers in other crops, including wheat (Kudoyarova et al., 2014), corn (Piromyou et al., 2011), mung bean (Ahmad et al., 2011), and peanut (Dey et al., 2004). Furthermore, the outstanding performance of P16, compared to that of P6, could be due to its siderophore production, which helps the plant in iron acquisition (Qi and Zhao, 2013), and its ACC-deaminase activity, which protects the plant from the deleterious effect of different stresses by suppressing ethylene synthesis (Barnawal et al., 2014).

In the second part of our study, the interactive effect of Si and rhizobacterial inoculation on salt tolerance of mung bean was studied under natural field conditions using saline irrigation. Our results revealed that gaseous exchanges, such as stomatal conductance and transpiration rate, are adversely affected by salt stress. The reduction in stomatal conductance may be due to the salinity causing damage to the surface of stomata (Gunes et al., 2007; Acosta-Motos et al., 2015). The reduction in transpiration rate may be attributed to reduction in stomatal conductivity, which occurs because the stomata close to maintain cell turgor under osmotic stress (Chedlia et al., 2007). In our study, plants treated with Si and rhizobacterial inoculation exhibited increased stomatal conductance and transpiration rate under saline irrigation conditions, which likely indirectly improved the photosynthetic rate, leading to increased growth, and yield of mung bean (Ahmad et al., 2012, 2013). Our results are supported by those of Farshidi et al. (2012), who noted increased transpiration rates and stomatal density in canola in response to Si application under salt stress. More recently, Shi et al. (2013) and Abbas et al. (2015) reported that Si application increased photosynthetic rate, stomatal conductance, transpiration rate, and water use efficiency in tomato and okra, respectively, under salt stress. Similarly, bacteria have been reported to alleviate salinity stress by improving stomatal conductance and transpiration in sweet pepper (del Amor and Cuadra-Crespo, 2012) and mung bean (Ahmad et al., 2013).

A decrease in relative leaf water content is a typical plant response to osmotic stress (Fahad et al., 2015). In our study, RWC increased for the Si and bacterial inoculated treatments under salt stress. This is an indication of enhanced water uptake due to the Si foliar spray and/or bacterial inoculation under saline conditions. Similar to our results, application of Si improved the RWC in salt-stressed wheat (Bybordi, 2014), sorghum (Yin et al., 2013; Liu et al., 2015), and tomato (Li et al., 2015). Furthermore, Nadeem et al. (2010) and Ahmad et al. (2013) noted increased RWC of wheat and mung bean, respectively, due to bacterial inoculation under saline conditions. Bacterial inoculation may reduce the inhibitory effect of salt stress on the roots and aid in the develop of more effective root systems, which could help plants absorb relatively more water from deeper soil under stress conditions (Marulanda et al., 2010).

Salinity stress damages the cell membrane due to increased EL (Tuna et al., 2008). Our results showed that EL increased with increasing salinity levels, which indicates more cell membrane damage at higher salinity levels. However, Si and bacterial inoculation reduced EL, which likely resulted in less membrane damage. Similar results were noted by Tuna et al. (2008) and Haghighi and Pessarakli (2013), who reported that Si application decreased the EL in salt-stressed wheat and tomato, respectively, from that in non-Si treated salt stressed plants. In addition, PGPR inoculation reduced cell membrane damage in salt-stressed maize (Marulanda et al., 2010) and cucumber (Kang S. M. et al., 2014), from that in the uninoculated control.

In the current study, Si foliar spray and bacterial inoculation increased the photosynthetic pigments (total chlorophyll, Chl a, Chl b, and carotenoids content) of mung bean under saline conditions. Similar to our results, Si applications have been noted to increase photosynthetic pigments in important agricultural crops under saline conditions, including wheat (Tuna et al., 2008; Chen et al., 2014), canola (Farshidi et al., 2012), soybean (Lee et al., 2010), and tomato (Haghighi and Pessarakli, 2013). In addition, the ameliorating effect of PGPR inoculation on chlorophyll content was observed in canola (Jalili et al., 2009), wheat (Nadeem et al., 2010), mung bean (Ahmad et al., 2013), pea (Barnawal et al., 2014), and soybean (Kang S. et al., 2014). Wu et al. (2015) observed a positive correlation between leaf tissue chlorophyll content and overall plant salinity tolerance. An elevated synthesis of chlorophyll content in plants leads to higher photosynthetic rate and starch production, which might support plant growth improvement under stress conditions (Kang S. et al., 2014).

Our results revealed that salinity significantly decreased the growth and yield of mung bean. However, Si and bacterial inoculation alleviated the harmful effects of salinity on growth and yield of mung bean. This growth improvement in the presence of Si under saline stress might be correlated with the maintenance of plant-water relations and membrane stability, increased photosynthetic pigments, and modification of gas exchange attributes (Haghighi and Pessarakli, 2013; Shi et al., 2013; Liu et al., 2015). In a previous study, Si application improved the shoot length and plant dry weight in salt stressed soybean (Lee et al., 2010). Other previous studies have also reported beneficial effects of Si on growth and yield of various cultivated plant species under salt stress conditions (Romero-Aranda et al., 2006; Farshidi et al., 2012; Yin et al., 2013; Chen et al., 2014). In addition, PGPR are widely reported to improve growth and development of different crops under salt stress conditions, both in axenic and field studies (Nadeem et al., 2010, 2013; Ahmad et al., 2011, 2012, 2013; Barnawal et al., 2014; Kang S. et al., 2014). The enhanced mung bean growth under saline conditions, due to bacterial inoculation, might be attributed to bacterial IAA activity, which has a tremendous effect on root growth, and water and nutrient absorption from a greater soil volume (Principe et al., 2007). Likewise, bacterial ACC-deaminase activity likely diluted the effect of salinity on plant growth by lowering the accelerated synthesis of ethylene, which inhibits root growth (Ahmad et al., 2011). This might be due to the hydrolysis of ACC, the immediate precursor of ethylene in plants (Yang and Hoffman, 1984), into ammonia and α-ketobutyrate (Klee et al., 1991) by the rhizobacterial strain carrying ACC-deaminase enzyme activity. Another possible reason for the enhanced growth from the P16 inoculation is the exopolysaccharide activity of bacteria that bind the sodium, owing to its cementing properties, and decrease the availability of Na^+^ for plant uptake (Ashraf et al., 2004; Kohler et al., 2006; Nadeem et al., 2010). Our results are consistent with the findings of Martínez et al. (2015), who noted significant improvement in root and shoot dry weight of alfalfa under salt stress due to bacterial inoculation. Although the combined application of Si and PGPR for improving plant growth under saline conditions has not been previously reported, the improved growth and yield of mung bean might be due to a synergistic effect of Si and PGPR under salinity stress. However, Garg and Bhandari (2015) reported that combined application of Si and arbuscular mycorrhiza complemented each other to improve the growth and biomass production of *Cicer arietinum* under salt stress.

Production of small and thick leaves (i.e., a reduction in leaf surface area) under salinity stress has been recognized as an adaptation strategy to reduce water loss (Acosta-Motos et al., 2015). In the current study, the leaf areas were higher for plants treated with Si and rhizobacteria than for untreated plants. An increase in leaf area after the application of Si has also been reported in canola (Farshidi et al., 2012), sorghum (Yin et al., 2013), and wheat (Chen et al., 2014). It has also been reported that PGPR-treated *Cucumis sativus* plants counteract the adverse effects of salt stress by producing low levels of abscisic acid, which results in a significant increase in leaf area (Kang S. M. et al., 2014). A significant increase in leaf area was also noted for bacterized maize under stress conditions (Naveed et al., 2014).

In our study, foliar spray of Si and bacterial inoculation improved the STI of mung bean. The improvement in the STI might be due to the Si-mediated alteration of the plants' physiology (Shi et al., 2013; Abbas et al., 2015), which led to higher biomass yield. The increased STI of mung bean is correlated with the bacterial production of exopolysaccharides, which might have altered the rhizosphere of the mung beans by creating a biofilm on the root surface, resulting in improved water and nutrient availability. This proposed explanation is consistent with the results of Hussain et al. (2014), who reported better drought tolerance index for inoculated maize seedlings than for an uninoculated control.

In conclusion, the present study comprehensively quantified the effect of salinity on the physiology, growth, and yield of mung bean. Moreover, foliar sprays of Si (1 and 2 kg ha^−1^) and two promising PGPRs (*E. cloacae* and *B. drentensis*) from the harsh natural saline conditions of Saudi Arabia were assessed for their ability to mitigate the adverse effects of salinity. The present study revealed that 2 kg Si ha^−1^ with the PGPR strain *B. drentensis* was the most effective combination for improving gaseous exchange, water relations, photosynthetic pigments, growth, and seed yield for mung bean under saline irrigation conditions. Moreover, the combined use of Si and PGPR showed an additive effect and represents an improved strategy for the amelioration of salinity stress in mung bean. Our research suggests that the combined use of Si and PGPR in agricultural is a sustainable strategy for the alleviation of salinity stress in mung bean. Further studies are required to explore the effect of Si on microbial gene expression and plant biochemistry, and to test whether the application of Si and PGPR might be a general strategy to improve stress tolerance of other crops.

## Author contributions

SM, ID, SA, and SA designed and executed the experiments. MM, SM, and SA carried out the lab work regarding chlorophyll measurement, relative water content, and electrolyte leakage. MY and ID were involved in rhizobacteria isolation, characterization and identification. SM and ID performed statistical analysis and wrote the manuscript. HH, SA, and ZA provided throughout technical support and helped in concluding the manuscript.

### Conflict of interest statement

The authors declare that the research was conducted in the absence of any commercial or financial relationships that could be construed as a potential conflict of interest.
